# Sir Benjamin Brodie

**Published:** 1859-03

**Authors:** 


SIR BENJAMIN BRODIE.—An unfounded report in one of the foreign periodicals soon gained currency that this distinguished member of our profession had been made a member of the House of Peers. This report has, however, served the purpose of awakening the medical men of London to the conviction that they are entitled to a representative in the Upper House, and they will, no doubt, take action upon the subject, and refer it to Lord Derby, who will give the question an impartial hearing. Without this advancement, Sir Benjamin Brodie now has a higher position than has ever before been held by a British surgeon, having been recently elected President of the Royal Society, and President of the Medical Council. We hope that he may attain this higher honor, to which his high scientific and literary attainments most certainly entitle him.

